# Desaturation-Induced Brightness in Face Color Perception

**DOI:** 10.1177/2041669519854782

**Published:** 2019-06-11

**Authors:** Hitomi Shimakura, Katsuaki Sakata

**Affiliations:** Shiseido Global Innovation Center, Japan; Joshibi University of Art and Design, Japan

**Keywords:** face, color, brightness, saturation, whiteness, inverted Helmholtz–Kohlrausch effect

## Abstract

The distinctiveness of perception of face from nonface objects has been noted previously. However, face brightness is often confounded with whiteness in the beauty industry; few studies have examined these perceptual differences. To investigate the interactions among face color attributes, we measured the effect of saturation on brightness and whiteness in both uniform color patches and face images to elucidate the relationship between these two perceptions. We found that, at constant luminance, a uniform color patch looked brighter with an increase in saturation (i.e., the Helmholtz–Kohlrausch effect occurred), while in contrast, brightness of a facial skin image looked less bright with increased saturation (i.e., contrary to the Helmholtz–Kohlrausch effect), which suggested this interaction of color attributes was influenced by top-down information. We conclude that this inverse effect of saturation on brightness for face images is not due to face recognition, color range of the skin tone, the luminance distribution, or recognition of human skin but due to the composite interactions of these facial skin factors in higher order recognition mechanisms.

## Introduction

### Facial Attractiveness and Its Perception

Humans are social animals that use facial expressions to recognize and communicate many types of information. In particular, facial attractiveness is considered important for humans to obtain a plethora of social information, such as reliability and familiarity of individuals (Kiiski, Cullene, Clavin, & Newell, 2016; Pei & Meng, 2018; Thiruchselvam, Harper, & Homer, 2016). Furthermore, it has been reported that perception of an attractive face is an unconscious process (Hung, Nieh, & Hsieh, 2016), which involves a relationship between efficient facial processing and the neural system’s preference for this type of stimulus (Tagai, Shimakura, Isobe, & Nittono, 2017). Therefore, it is believed that facial attractiveness is fundamentally important for humans (Haist & Anzures, 2016; Ohmann, Stahl, Mussweiler, & Kedia, 2016), and it has been reported that skin tone strongly influences not only perception of emotional expressions (Minami, Nakajima, & Nakauchi, 2018) but also facial attractiveness (Coetzee et al., 2012). It has also been reported that incremental increases in redness (CIE a*) are associated with perceptions of a healthier face (Stephen, Law Smith, Stirrat, & Perrett, 2009; Thorstenson, Pazda, Elliot, & Perrett, 2016).

### Perception of Facial Skin Tone

In East Asian countries, people tend to have a greater preference for lighter skin tone and more whiteness, and terms such as whiteness, lightness, brightness, and fairness are used in the appraisal of facial attractiveness worldwide (Hunter, 2002; Kawada et al., 2002; Sahay & Piran, 1997; Setchell, 2005; Wade, Romano, & Blue, 2004; Yoshikawa, Kikuchi, Yaguchi, Mizokami, & Takata, 2012). In the cosmetics industry, words and phrases are widely used in this respect, such as radiant, even skin tone, uniform tone, and clarity. These words might be related to perceived brightness and lightness of the skin. In addition, luminance is often used as an independent variable to measure not only lightness but also perceived whiteness of a face.

Brightness is defined as “the attribute of a visual sensation according to which a given visual stimulus appears to be more or less intense” (Wyszecki & Stiles, 1982, p. 487). The term whiteness is often used to describe lightness of skin tone, where “lightness” is defined as “the attribute of a visual sensation according to which the area in which the visual stimulus is presented appears to emit more or less light in proportion to that emitted by a similarly illuminated area perceived as a ‘white’ stimulus” (Wyszecki & Stiles, 1982, p. 487). The fact that whiteness can be used to refer to brightness in the beauty industry may show the uniqueness of face color perception. Whiteness perception of facial skin tone is reported to be affected by decreasing saturation at constant luminance, and whiteness of a facial skin image, but not of a color patch image, is affected by both its saturation and hue (Yoshikawa et al., 2012). Further, many reports suggest that face perception involves several unique performance strategies (Bindemann, Burton, Hooge, Jenkins, & Haan, 2005; Ro, Russell, & Lavie, 2001; Tarr & Cheng, 2003). Moreover, previous studies have shown the uniqueness of face perception, where upright facial images retain more attention than object images (Bindemann et al., 2005), change blindness is weaker for human facial images than for other common objects (Ro et al., 2001), and visual sensitivity for redness and yellowness of facial tone are greater than for those of color patches (Tan & Stephen, 2013). In addition, specific models of face perception have been proposed (Burton, Bruce, & Johnston, 1990). These studies suggest that our perception of facial images has unique characteristics (Tarr & Cheng, 2003). Therefore, in this study, we explored the uniqueness of face color perception by measuring and comparing brightness and whiteness of a human face and of a color patch.

## Experiment 1

### Aim

As brightness is determined by luminance for a given constant condition, we examined the effect of luminance on brightness and perceived whiteness of skin tone in face images.

### Methods

#### Participants

Participants were 20 females (mean age = 22.2 years old, *SD* = 2.8), with normal color vision as screened using Ishihara pseudo-isochromatic plates, and visual acuity, including corrected visual acuity, confirmed to be within the normal range.

The study protocol was approved by the ethics committees at the Shiseido Global Innovation Center and Joshibi University of Art and Design. All procedures conformed to the Declaration of Helsinki, and all participants provided written informed consent.

#### Stimuli

The stimuli were a pair of face images of an average Japanese female in her 30s, which were produced by superimposing several Japanese female face images with three levels of lightness, one level of which simulated that of the average skin tone of Japanese female face (L* = 65, a* = 10, b* = 18; Hamada, Mizokami, Kikuchi, Yaguchi, & Aizu, 2018), while the other two levels of luminance were adopted to compare the effects of lightness levels and to control for Japanese preferences for brighter faces (L* = 70, and L* = 75, a* = 10, b* = 18). All stimuli were presented on an organic electroluminescent display monitor (SONY) controlled by a PC (HP), against a uniform grey background, Y = 34.2, (*x*, *y*) = (0.31, 0.33). Each stimulus face size was approximately 15 degrees (height) × 12 degrees (width), assuming a real face would be viewed at a distance of 0.8 m.

#### Procedure

The experiment was carried out in a dark room. The participant’s head was fixed on a chin rest throughout the experimental trials. Each trial was started after 5 minutes of monitor-background grey adaptation.

**Figure 1.**
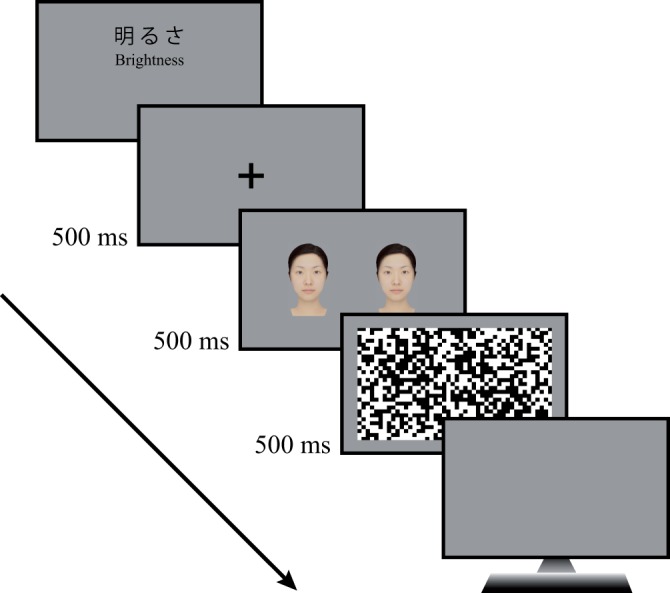
The time course of stimulus presentation. As shown here, 500 ms presentation of a fixation cross (+) at the center of the screen was followed by a pair of face images on a grey background for 500 ms, followed by a random dot mask for 500 ms.

The participants completed a two-alternative forced choice task by indicating which of the two stimuli, left or right, was brighter (whiter) and inputting their responses by pressing alternative keys on the partially restricted keypad. A fixation cross appeared in the center of the screen for 500 ms after presentation of a “brightness” cue (明るさ) in brightness judgment sessions or a “whiteness” cue (白さ) in whiteness judgment sessions. Next, a pair of face images were presented for 500 ms, then a black-and-white random pattern for 500 ms to mask the subsequent afterimages, as shown in Figure 1. We instructed participants to make judgments based on a whole face image instead of only a part of the face. Sixty trials were conducted (3 Lightness Levels × 10 Repetitions × 2 Perceived Attributes: brightness or whiteness).

### Results

**Figure 2.**
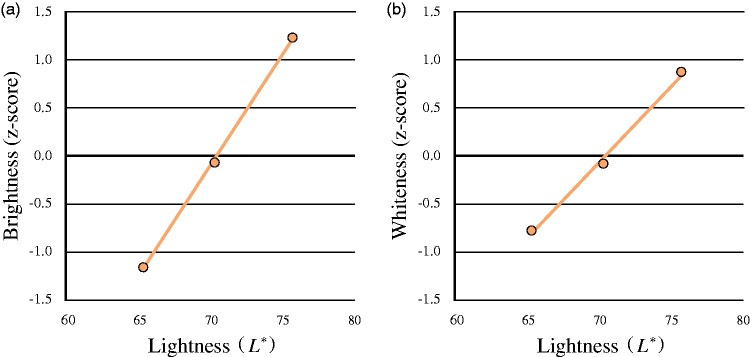
Perceived brightness and whiteness by face luminance level. The *z*-scored brightness and whiteness increased as the luminance of the images increased. (a) Mean perceived brightness and (b) mean perceived whiteness changed per level of face luminance.

We used the CIE L*a*b* system to characterize color stimuli, consistent with a prior study (Sanchez & Fairchild, 2002). The observers’ perceived brightness and whiteness were calculated via a custom VBA program, using the Thurstone Case V scaling technique to construct the pair-comparison scale (Asparouhov & Muthen, 2010; Maydeu-Olivares & Bockenholt, 2005; Mosteller, 1951; Thurstone, 1927). Figure 2 shows the *z*-scored scale values of brightness and whiteness of the facial image for different levels of lightness induced by manipulation of luminance. We used IBM SPSS Statistics (IBM-SPSS, Chicago, IL) to conduct the analyses. A one-way repeated-measures analysis of variance (ANOVA) showed that both brightness and whiteness significantly increased with higher lightness—brightness: *F*(2, 38) = 587.81, *p* < .0001; whiteness: *F*(2, 38) = 89.78, *p* < .0001—which indicated that face color was perceived as brighter and whiter with increased lightness of face images with the same chromaticity coordinates. That is, the observers’ perceived whiteness was highly associated with lightness as well as brightness.

### Discussion

The similar tendency in all observers’ data and monotonic increasing of both brightness and whiteness with luminance suggest high reliability of the experimental results. Since all face stimuli used in the experiment were of the same chromaticity—that is, the same hue and saturation—the results of the experiment indicated that the luminance of the face images induced perceptions of both brightness and whiteness. This result is consistent with previous studies using nonface stimuli (Ikeda, Mizokami, Nakane, & Shinoda, 2002), which showed that the correlation of whiteness with luminance is not a unique feature of facial tone stimuli when chromaticity is held constant.

In Experiment 2, we examined the effect of saturation on brightness and whiteness of a face image and a color patch, and compared this effect with the effect of luminance, to determine if face perception has unique characteristics.

## Experiment 2

Since both brightness and whiteness increased with L* in Experiment 1, we could not identify the differences between brightness and whiteness in face images. Consequently, we tested the alternative idea affecting brightness and whiteness, namely, saturation at constant lightness, because the distance from white would decrease with a decrease in saturation. As brightness is known to increase with saturation through the Helmholtz–Kohlrausch effect (H-K effect), we confirmed the effect of saturation on whiteness as well as brightness.

### Aim

The effects of saturation on face brightness and whiteness were measured and compared with the effects of saturation on a color patch. If color perception of a face differed from that of a color patch because of face color constancy, we might obtain different results for face perception brightness constancy than for color patch brightness constancy (Tan & Stephen, 2013).

### Methods

#### Participants

Participants were 21 females (mean age = 23.8 years old, *SD* = 3.1) who were screened for normal color vision using the same procedure as in Experiment 1.

#### Stimuli

**Figure 3.**
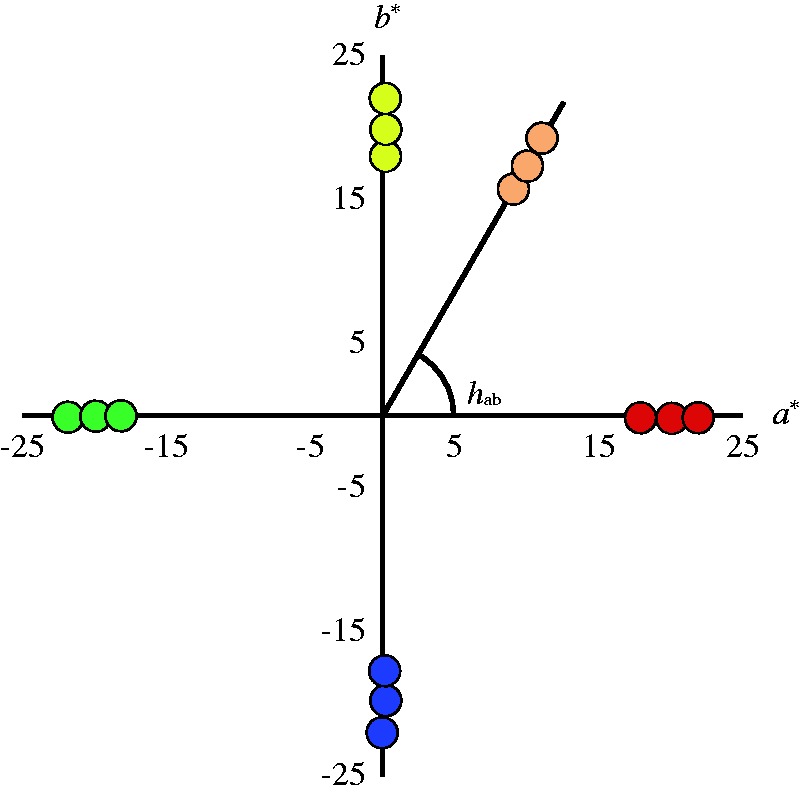
CIE a*b* chromaticity coordinates of stimuli. The uniform color patches had five variations in hue: *h*_ab_ = 0 (Red), *h*_ab_ = 60 (the average skin tone of Japanese female face), *h*_ab_ = 90 (Yellow), *h*_ab_ = 180 (Green), and *h*_ab_ = 270 (Blue).

The stimuli were uniform color patches at 2 degrees of visual angle, in addition to the same face images used in Experiment 1. The chromaticity coordinates of the face stimuli and the uniform patches are shown in Figure 3. For the face stimuli, the hue was *h*_ab_ = 60 and the saturation was C*_ab_ = 20, which was that of the average skin tone of Japanese female face shown in previous experiment. Two variations of saturation stimuli, higher (C*_ab_ = 22) and lower (C*_ab_ = 18), were also adopted for the same hue. The uniform color patches had five variations in hue: *h*_ab_ = 0, *h*_ab_ = 60, *h*_ab_ = 90, *h*_ab_ = 180 and *h*_ab_ = 270 and the saturations were the same as for the face stimuli. The luminance was presented at 34.2 cd/m^2^ simulating as L* = 65 for all stimuli, which represents the average luminance of a Japanese female face.

#### Procedure

All experimental trials were conducted under the same conditions as in Experiment 1. Each participant completed 360 trials with three combinations of 3 Saturation Levels × 10 Repetitions × 2 Perceived attributes for the face image, and three combinations of 3 Saturation Levels × 5 Hues × 10 Repetitions × 2 Perceived attributes for the color patch.

### Results

**Figure 4.**
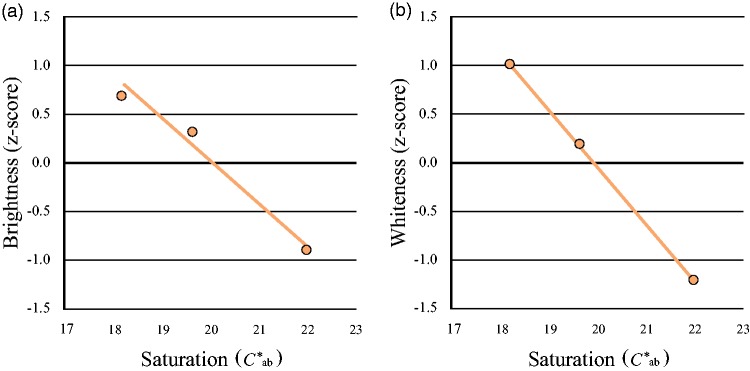
Perceived brightness and whiteness with saturation of the face image. The *z*-scored brightness and whiteness increased as saturation of the face image decreased. (a) Mean perceived brightness and (b) mean perceived whiteness changed per level of saturation of the face image.

**Figure 5.**
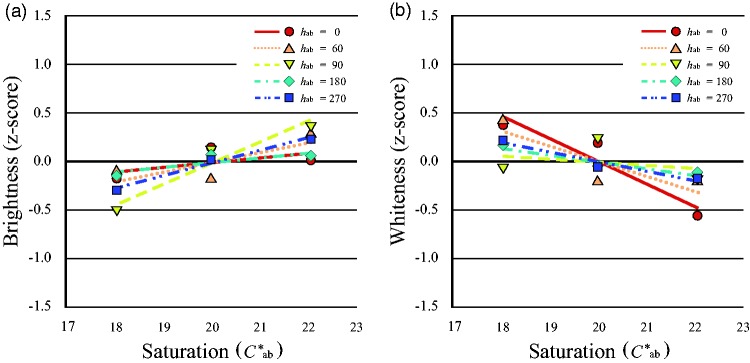
Perception of brightness and whiteness changed with the level of saturation of the uniform color patch. As saturation of the uniform color patch increased, brightness perception increased but whiteness perception decreased. (a) The *z*-scored mean perceived brightness increased per level of saturation of the uniform color patch while (b) *z*-scored mean perceived whiteness decreased per level of saturation of the uniform color patch. Continuous line with ● shows brightness or whiteness for *h*_ab_ = 0, dotted line with ▲ for *h*_ab_ = 60, dashed line with ▼ for *h*_ab_ = 90, dashed dotted line with ♦ for *h*_ab_ = 180, and dashed double-dotted line with ■ for *h*_ab_ = 270.

Participants’ responses were analyzed to calculate scale values using the Thurstone Case V scaling technique, as in Experiment 1. Figure 4 shows the *z*-scored scale values of brightness and whiteness for all participants against the saturation of the face image; ANOVAs were conducted to compare the effects of the various factors. Both brightness and whiteness in face images decreased with saturation increment at constant luminance—brightness: *F*(2, 40) = 96.27, *p* < .0001; whiteness: *F*(2, 40) = 210.46, *p* < .0001. Therefore, decrease in saturation resulted in perception of the face as both brighter and whiter. In turn, Figure 5(a) shows the *z*-scored brightness of the uniform color patches with different levels of saturation for each of the five hues. For every hue, brightness increased with increasing saturation—*h*_ab_ = 0: *F*(2, 40) = 3.68, *p* = .034; *h*_ab_ = 60: *F*(2, 40) = 7.68, *p* = .002; *h*_ab_ = 90: *F*(2, 40) = 28.05, *p* < .0001; *h*_ab_ = 180: *F*(2, 40) = 4.92, *p* = .012; *h*_ab_ = 270: *F*(2, 40) = 15.12, *p* < .0001. No significant differences were observed according to hue variation, which suggests that the H-K effect was not due to human skin tone. In contrast, in all participants whiteness of the uniform color patch decreased with saturation for all variations of hue—*h*_ab_ = 0: *F*(2, 40) = 56.09, *p* < .0001; *h*_ab_ = 60: *F*(2, 40) = 20.48, *p* < .0001; *h*_ab_ = 90: *F*(2, 40) = 12.20, *p* < .0001; *h*_ab_ = 180: *F*(2, 40) = 6.08, *p* = .005; *h*_ab_ = 270: *F*(2, 40) = 13.55, *p* < .0001; Figure 5(b). We could not confirm any superiority of an approximately human-skin hue (*h*_ab_ = 60).

**Figure 6.**
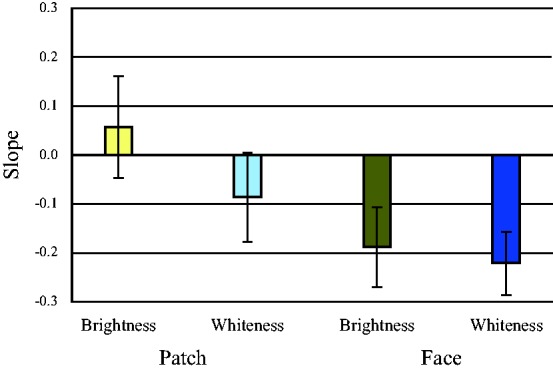
The slope of brightness and whiteness for the average of skin tone. The absolute slopes for the face stimuli were both negative and greater than those for the uniform color patches for both brightness and whiteness. The error bars represents ±1 standard deviation.

Figure 6 shows a comparison of the slope of brightness and whiteness functions of the face stimuli and the uniform color patches at *h*_ab_ = 60. The results of one-way ANOVA to the absolute values of the slopes showed that the slopes of both brightness and whiteness of the face were steeper than those of the uniform color patches—brightness: *F*(1, 20) = 22.60, *p* < .0001, whiteness: *F*(1, 20) = 76.25, *p* < .0001. The whiteness slope did not differ from that of brightness—*F*(1, 20) = 6.32, *p* = .021 for face stimuli; *F*(1, 20) = .76, *p* = .394 for the uniform color patches.

### Discussion

Brightness of the uniform color patches increased with saturation at constant luminance in this experiment, namely, the H-K effect, which occurs when a chromatic color appears brighter than an achromatic color of the same luminance (Wyszecki & Stiles, 1982). Considering proposed models of the H-K effect (Corney, Haynes, Rees, & Lotto, 2009; Withouck et al., 2013), this result with uniform color patches could replicate the H-K effect. In contrast, brightness of the face stimulus increased as saturation decreased, at constant luminance; that is, an inverted H-K effect occurred. This finding suggests that brightness perception of face images involves a mechanism that differs from that for uniform color patches. However, a previous study reported that the “feeling of brightness” of facial photographs increased as the saturation increased, and they called this the H-K effect (Shizume, Ohashi, Takamatsu, & Shimodaira, 2014). Similar results were also obtained for nonface images in their experiment, which suggests that the H-K effect may be observed not only for face images but also for other images. However, the authors varied the whole color composition of the image, including the background, in their stimuli in their experiment, whereas we only changed the facial tone; therefore, their result could reflect the effect of color constancy. This might explain the inconsistent findings between the previous study showing the H-K effect and our result showing an inverted effect for face images.

Since both the H-K effect for the uniform color patches and the inverted H-K effect for the face stimuli were common to all our participants, our results suggest this visual effect is robust. Since the appearance of the H-K effect or the inverted H-K effect depended on the type of stimulus, this brightness perception of face color affected by saturation is consistent with a higher order cognitive process that alters brightness depending on the image type. As whiteness was affected by luminance with constant chromaticity in Experiment 1, and by saturation with constant luminance in both the facial images and the uniform color patches in this experiment, whiteness might be correctly perceived depending on distance of the image chromaticity from white in the CIE L*a*b* uniform color space, regardless of stimulus type. Thus, the uniqueness of face color perception is not with respect to face whiteness but face brightness.

Since both brightness and whiteness of face images showed monotonic decreasing with saturation, the whiter face could be brighter regardless of the constant luminance. By definition, whiteness is dissimilar to brightness; that is, whiteness is an attribute of light reflectance, while brightness is an attribute of light intensity (Kobayashi, Matsushita, & Morikawa, 2018; Schirillo, Reeves, & Arend, 1990). In our results, face whiteness behaved similarly to uniform patch whiteness with changes in saturation, while face brightness behaved in the opposite manner to uniform color patch brightness. This could be a unique aspect of face color perception, which may involve feedback information from a higher order cognitive process.

Given that the perceived scale value distance calculated by the probability of judgment was represented as the slope of the regression line in the figure, the greater slope of brightness and whiteness for the face stimulus compared to the uniform color patch for *h*_ab_ = 60 suggests high sensitivity for face color, and ease of distinguishing various functional aspects of face color. Furthermore, the greater slope of perceived whiteness compared to brightness suggests that whiteness was easier to judge than brightness for both face stimuli and uniform color patches. Since the independent variables were step levels of saturation on which whiteness depended, whiteness judgment might be easier than brightness judgment at a constant luminance.

For the uniform color patches, the colors of which were any of five hues to enable investigation of the difference between skin tone and other hues, the results showed no difference in saturation effects on either brightness or whiteness for any hue. However, previous research has shown that the degree of the H-K effect varies with hue variations (Corney et al., 2009). Similar to our results with uniform color patches, the previous study showed that brightness appearance increased as saturation increased at constant luminance, and the effect was smallest at 450 nm and largest at 570 nm (Uchikawa, Uchikawa, & Kaiser, 1984). These studies have not shown the superiority of human facial tone when judging either brightness or whiteness, and we could not find a significant difference between the result at *h*_ab_ = 60 and the other hues. Therefore, neither the decreasing brightness with saturation nor the high sensitivity of judgments are due to the skin tone itself.

These results suggest that brightness and whiteness might be conflated only for face stimuli. Since this experiment used only two types of images (face stimuli and uniform color patches), it was difficult to clarify the factors affecting face color perception. Factors that could conceivably distinguish the two types of stimuli include shape, texture, presence of parts, contrast variation, and so on. Therefore, in Experiment 3, we examined the degree of “faceness,” that is, stimulus recognizability as a face or its proxy, as an influence on the effect of saturation on face brightness or whiteness perception.

## Experiment 3

Factors underlying differences between the effect of saturation for the uniform color patches and those for the face stimuli in Experiment 2 could include the size and shape of the stimulus, hue, recognition of human skin, recognition of a face, recognition of a face image, and uniformity of contrast. Thus, we measured and analyzed the influence of these factors on the effects of saturation on brightness and whiteness judgments. If the results for the face stimuli in Experiment 2 were uniquely due to face recognition, then the H-K effect would be observed even for a face image that was not recognized as a face. In contrast, if the stimulus was perceived as a face, the inverted saturation effect would be observed. Since the face stimulus was 15 degrees (height) × 12 degrees (width) and the color patch was a 2-degree diameter disk in the previous experiments, we adopted a uniform color patch stimulus of the same size as the face stimulus. Therefore, we examined why a decrease in brightness with saturation may be perceived as an increase, contingent on three factors: stimulus size, skin recognition, and degree of face recognition. Furthermore, the effect of hue was also examined. We assessed the effect of skin tone compared to four other hues in face images and in some variations of uniform color patches.

### Aim

To identify unique characteristics of face color perception, we analyzed possible factors underlying the discrepancy in the effect of saturation on brightness perception in a uniform color patch versus a face image.

### Methods

#### Participants

Twenty females (mean age = 24.4 years old, *SD* = 6.4) with normal color vision (screened using the same procedure as in Experiments 1 and 2) participated in this experiment.

#### Stimuli

**Figure 7.**
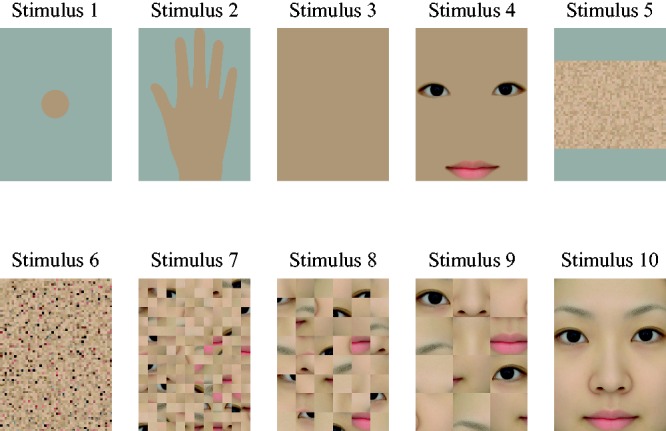
Stimulus images. Stimulus 1 to Stimulus 4 were “uniform” color patches: a 2-degree disk (Stimulus 1), hand-shaped (Stimulus 2), a patch of the same size as the face images (Stimulus 3), a patch of the same size as the face images with eyes and mouth (Stimulus 4). Stimulus 5 to Stimulus 10 were “face” images: scrambled into 30 by 30 pixel patches without high contrast dark parts (Stimulus 5), scrambled into 30 by 30 pixel patches (Stimulus 6), scrambled into 120 by 120 pixel patches (Stimulus 7), scrambled into 240 by 240 pixel patches (Stimulus 8), scrambled into 480 by 480 pixel patches (Stimulus 9), and an original “face” image (Stimulus 10).

As shown in Figure 7, stimuli were four uniform stimuli (Stimulus 1 to Stimulus 4) and six face stimuli (Stimulus 5 to Stimulus 10). The uniform stimuli were as follows: Stimulus 1, “disk,” which was the 2-degree uniform color patch used in Experiment 2; Stimulus 2, “hand,” a hand-shaped color patch with a grey background; Stimulus 3, “rectangular patch,” which was a uniform patch of the same size as the face stimulus; and Stimulus 4, “rectangular patch with eyes and mouth”. Stimulus 3 and Stimulus 4 were the same size as the face stimulus. The face stimuli were as follows: Stimulus 10, “face,” which was the same face stimulus used in Experiments 1 and 2; Stimulus 9, “scrambled face (480 px × 480 px),” which was a scrambled image of Stimulus 10; Stimulus 8, “scrambled face (240 px × 240 px)”; Stimulus 7, “scrambled face (120 px × 120 px)”; Stimulus 6, “scrambled face (30 px × 30 px)”. In addition, there was Stimulus 5, “scrambled face without dark parts (30 px × 30 px),” which excluded high-contrast dark areas, such as the eyebrows, eyes, nose, and mouth from Stimulus 6. Five different scrambled images for each of Stimulus 5 to Stimulus 9 were used with every saturation to avoid potential bias effects due to image contrast. All stimuli were generated with five hues (*h*_ab_ = 0, *h*_ab_ = 60, *h*_ab_ = 90, *h*_ab_ = 180, *h*_ab_ = 270) with three saturations and were presented on a PC-controlled monitor as in Experiments 1 and 2.

#### Procedure

The procedure was the same paired comparison method used in Experiments 1 and 2, except for the stimulus images. Since the results of a pilot study were stable, the participants were asked only for brightness assessments of Stimulus 1 (“disk”) to reduce their burden. Therefore, the total number of trials was 2850: three combinations of 3 Saturation Levels × 1 Perceived Attribute (brightness) × 5 Hues × 10 Repetitions = 150 trials for Stimulus 1; and three combinations of 3 Saturation Levels × 2 Perceived Attributes (brightness and whiteness) × 5 Hues × 10 Repetitions × 9 Images (Stimulus 2 to Stimulus 10) = 2700 trials for Stimulus 2 to Stimulus 10.

### Results

**Figure 8.**
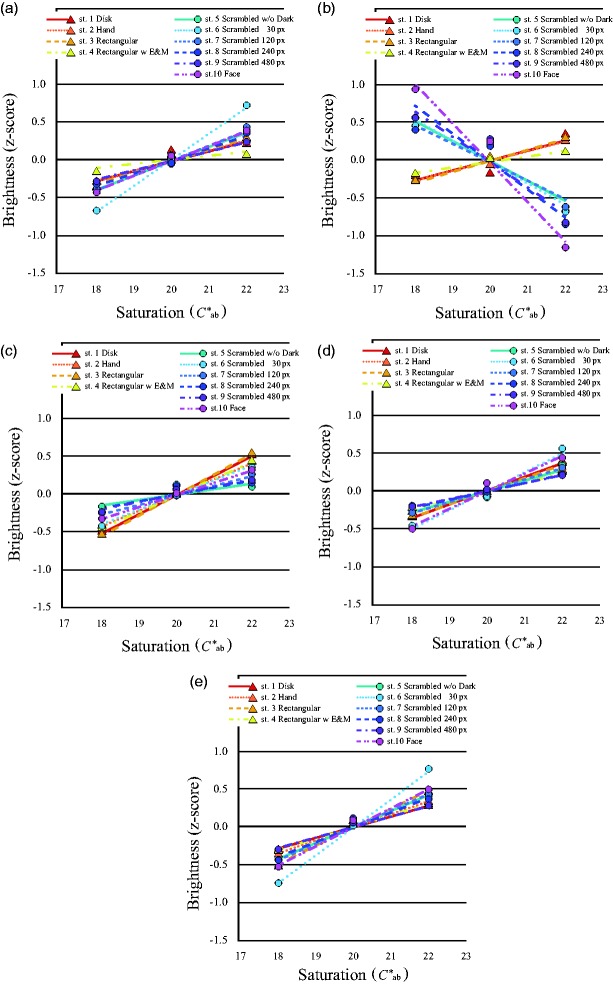
*Z*-scored mean brightness changed with saturation of each stimulus image. Mean brightness of each stimulus changed with saturation in each hue. (a) Stimulus images for *h*_ab_ = 0, (b) for *h*_ab_ = 60, (c) for *h*_ab_ = 90, (d) for *h*_ab_ = 180, and (e) for *h*_ab_ = 270. Only brightness of face images for *h*_ab_ = 60 decreased with saturation. ▲ with continuous line shows Stimulus 1, ▲ with dotted line shows Stimulus 2, ▲ with dashed line shows Stimulus 3, ▲ with dashed double-dotted line shows Stimulus 4, ● with continuous line shows Stimulus 5, ● with dotted line shows Stimulus 6, ● with short dashed line shows Stimulus 7, ● with long dashed line shows Stimulus 8, ● with dashed dotted line shows Stimulus 9, and ● with dashed double-dotted line shows Stimulus 10.

As in Experiments 1 and 2, participants’ responses were analyzed by the Thurstone Case V technique to construct the pair comparison scale. Figure 8(a) to (e) shows the shift of brightness caused by saturation for each hue. Brightness increased with saturation, namely, the H-K effect was observed for all stimuli and all hues, except for the images originally generated from a human face image with skin tone. The inverted H-K effect was furthermore observed not only for the complete face image (Stimulus 10), but also for all scrambled images (Stimulus 5 to Stimulus 9), even though the images were unrecognizable. One-way ANOVAs of the probability of subjects’ alternative forced choice supported these results (Table 1).

**Table 1.** Results of One-Way ANOVAs for Brightness by Saturation.

Stimulus No.	***h*_ab_= 0**	***h*_ab_ = 60**	***h*_ab_ = 90**	***h*_ab_ = 180**	***h*_ab_ = 270**
1	*F*(2, 38) = 24.57***	*F*(2, 38) = 19.45***	*F*(2, 38) = 37.18***	*F*(2, 38) = 29.32***	*F*(2, 38) = 16.54***
2	*F*(2, 38) = 10.83***	*F*(2, 38) = 10.80***	*F*(2, 38) = 19.88***	*F*(2, 38) = 16.02***	*F*(2, 38) = 13.72***
3	*F*(2, 38) = 14.33***	*F*(2, 38) = 12.90***	*F*(2, 38) = 27.59***	*F*(2, 38) = 25.83***	*F*(2, 38) = 40.13***
4	*F*(2, 38) = 3.22 n.s.	*F*(2, 38) = 5.05*	*F*(2, 38) = 34.81***	*F*(2, 38) = 22.68***	*F*(2, 38) = 46.25***
5	*F*(2, 38) = 28.40***	*F*(2, 38) = 95.64***	*F*(2, 38) = 5.34**	*F*(2, 38) = 23.98***	*F*(2, 38) = 42.86***
6	*F*(2, 38) = 100.29***	*F*(2, 38) = 50.13***	*F*(2, 38) = 22.69***	*F*(2, 38) = 41.69***	*F*(2, 38) = 109.93***
7	*F*(2, 38) = 25.06***	*F*(2, 38) = 46.00***	*F*(2, 38) = 9.40***	*F*(2, 38) = 15.00***	*F*(2, 38) = 27.09***
8	*F*(2, 38) = 14.79***	*F*(2, 38) = 124.27***	*F*(2, 38) = 5.74**	*F*(2, 38) = 6.84**	*F*(2, 38) = 20.22***
9	*F*(2, 38) = 8.18**	*F*(2, 38) = 148.81***	*F*(2, 38) = 3.78*	*F*(2, 38) = 5.48**	*F*(2, 38) = 7.30**
10	*F*(2, 38) = 9.96***	*F*(2, 38) = 271.06***	*F*(2, 38) = 6.98**	*F*(2, 38) = 23.78***	*F*(2, 38) = 25.58***

* *p*<.05,**p<.01,***p<.001

**Figure 9.**
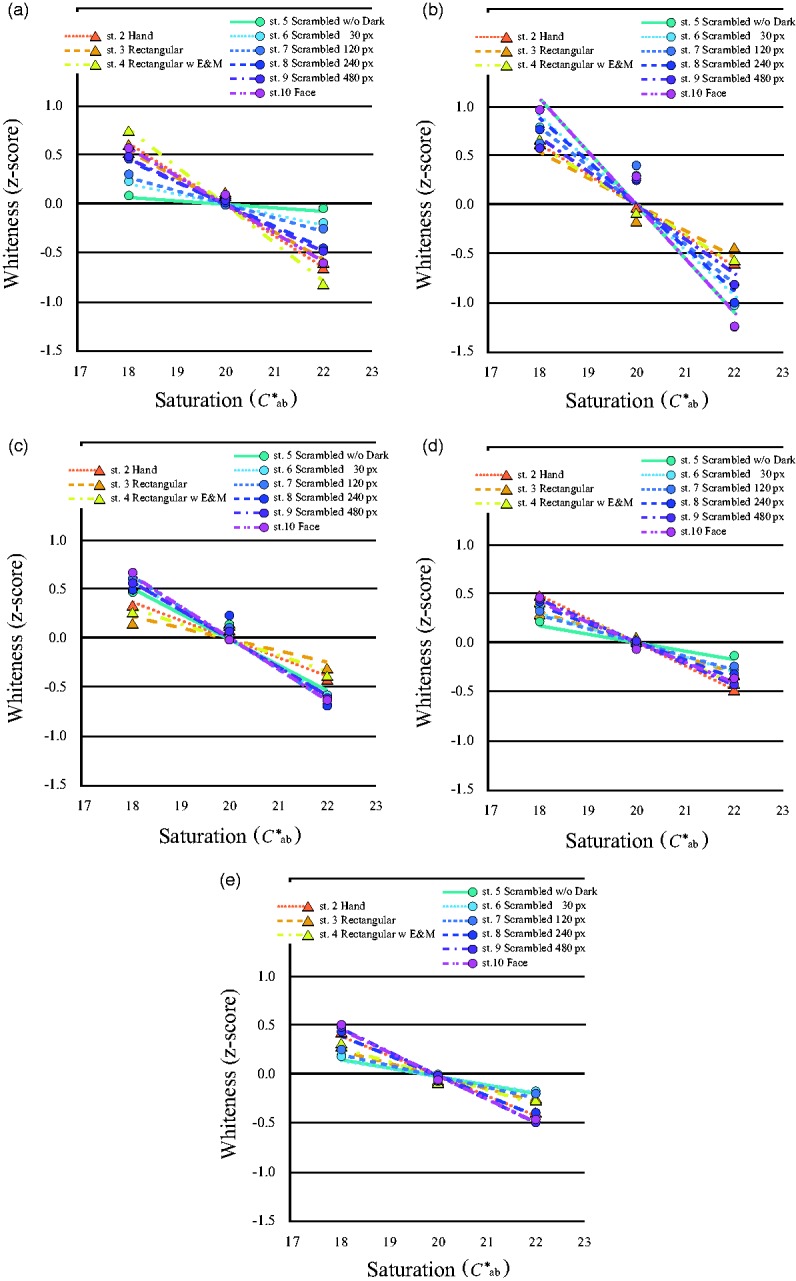
*Z*-scored mean whiteness changed with saturation of each stimulus image. Mean whiteness of each stimulus changed with saturation in each hue. (a) stimulus images for *h*_ab_ = 0, (b) for *h*_ab_ = 60, (c) for *h*_ab_ = 90, (d) for *h*_ab_ = 180, and (e) for *h*_ab_ = 270. Whiteness of all the stimulus images, even for face images or for *h*_ab_ = 60, decreased with saturation. ▲ with dotted line shows Stimulus 2, ▲ with dashed line shows Stimulus 3, ▲ with dashed double-dotted line shows Stimulus 4, ● with continuous line shows Stimulus 5, ● with dotted line shows Stimulus 6, ● with short dashed line shows Stimulus 7, ● with long dashed line shows Stimulus 8, ● with dashed dotted line shows Stimulus 9, and ● with dashed double-dotted line shows Stimulus 10.

In contrast, whiteness decreased with saturation for all stimuli and all hues (Figure 9 (a) to (e)). One-way ANOVAs indicated these decrements were statistically significant (Table 2).

**Table 2.** Results of One-Way ANOVAs for Whiteness by Saturation.

Stimulus No.	***h*_ab_= 0**	***h*_ab_ = 60**	***h*_ab_ = 90**	***h*_ab_ = 180**	***h*_ab_ = 270**
2	*F*(2, 38) = 78.21 ***	*F*(2, 38) = 86.87 ***	*F*(2, 38) = 15.27 ***	*F*(2, 38) = 42.64 ***	*F*(2, 38) = 27.72 ***
3	*F*(2, 38) = 58.09 ***	*F*(2, 38) = 50.02 ***	*F*(2, 38) = 5.47 **	*F*(2, 38) = 25.54 ***	*F*(2, 38) = 12.49 ***
4	*F*(2, 38) = 197.24 ***	*F*(2, 38) = 91.62 ***	*F*(2, 38) = 10.89 ***	*F*(2, 38) = 50.54 ***	*F*(2, 38) = 16.05 ***
5	*F*(2, 38) = 0.56 n.s.	*F*(2, 38) = 267.04 ***	*F*(2, 38) = 48.15 ***	*F*(2, 38) = 4.21 *	*F*(2, 38) = 4.69*
6	*F*(2, 38) = 5.54 **	*F*(2, 38) = 171.86 ***	*F*(2, 38) = 53.00 ***	*F*(2, 38) = 11.55 ***	*F*(2, 38) = 3.11 n.s.
7	*F*(2, 38) = 10.05 ***	*F*(2, 38) = 137.58 ***	*F*(2, 38) = 89.65 ***	*F*(2, 38) = 8.12 **	*F*(2, 38) = 5.63 **
8	*F*(2, 38) = 35.55 ***	*F*(2, 38) = 188.92 ***	*F*(2, 38) = 73.51 ***	*F*(2, 38) = 31.05 ***	*F*(2, 38) = 23.82 ***
9	*F*(2, 38) = 49.01 ***	*F*(2, 38) = 140.61 ***	*F*(2, 38) = 105.15 ***	*F*(2, 38) = 39.94 ***	*F*(2, 38) = 44.27 ***
10	*F*(2, 38) = 57.14 ***	*F*(2, 38) = 284.13 ***	*F*(2, 38) = 74.42 ***	*F*(2, 38) = 21.44 ***	*F*(2, 38) = 31.36 ***

*p<.05,**p<.01,***p<.001

**Figure 10.**
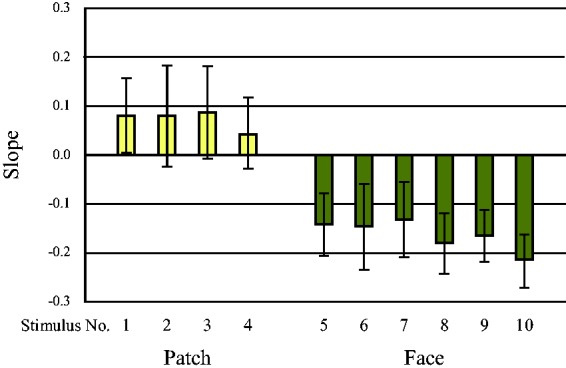
The slope for brightness of each stimulus for the average skin tone. All slopes for the face stimuli were negative and larger than those of the uniform color patches for brightness, and the slopes of the face images divided into larger patches (Stimulus 8, Stimulus 9, and Stimulus 10) were smaller than those for face images divided into smaller patches (Stimulus 5, Stimulus 6, and Stimulus 7). The error bars represents ±1 standard deviation.

The slopes of the regression lines for each stimulus were compared to investigate the sensitivity to brightness in the stimulus images of *h*_ab_ = 60 (Figure 10). The negative slopes for facial stimuli showed an inverse H-K effect, and the steeper slopes of the regression lines for brightness of the face stimuli, compared to those for the uniform stimuli, showed higher sensitivity for the face stimuli—*F*(1, 79) = 255.82, *p* < .0001. The sensitivity to whiteness was also greater for facial images than for uniform stimuli—*F*(1, 59) = 15.72, *p* < .0001. Moreover, the slopes of the regression lines of perceived whiteness were steeper than those of brightness for the uniform stimuli—*F*(1, 19) = 27.00, *p* < .0001 for Stimulus 2; *F*(1, 19) = 13.01, *p* = .002 for Stimulus 3; *F*(1, 19) = 48.16, *p* < .0001 for Stimulus 4—but not for all the face stimuli—*F*(1, 19) = 19.16, *p* < .0001 for Stimulus 5; *F*(1, 19) = 11.69, *p* = .003 for Stimulus 6; *F*(1, 19) = 8.36, *p* = .009 for Stimulus 7; *F*(1, 19) = 3.38, *p* = .082 for Stimulus 8; *F*(1, 19) = .005, *p* = .946 for Stimulus 9; *F*(1, 19) = .42, *p* = .523 for Stimulus 10—and these results were similar for the other hues. These results showed that brightness judgments differed from whiteness judgments for the less scrambled images and images of uniform patches; however, the sensitivities of these two types of judgments were similar if the images were recognizable as a human face. Since we could not find any significant differences for *h*_ab_ = 60 for the other hues, we could not confirm the particularity of human skin tone; however, the results showed a significant main effect of hue for each stimulus—*F*(4, 76) = 4.24, *p* = .004 for Stimulus 1; *F*(4, 76) = 5.79, *p* < .0001 for Stimulus 2; *F*(4, 76) = 2.64, *p* = .040 for Stimulus 3; *F*(4, 76) = 10.27, *p* < .0001 for Stimulus 4; *F*(4, 76) = 73.91, *p* < .0001 for Stimulus 5; *F*(4, 76) = 97.06, *p* < .0001 for Stimulus 6; *F*(4, 76) = 48.80, *p* < .0001 for Stimulus 7; *F*(4, 76) = 68.28, *p* < .0001 for Stimulus 8; *F*(4, 76) = 45.45, *p* < .0001 for Stimulus 9; *F*(4, 76) = 76.39, *p* < .0001 for Stimulus 10. Comparing the differences in hue for each whiteness slope, we also did not observe systematic effects of *h*_ab_ = 60, namely, human skin tone.

### Discussion

The monotonic increasing of brightness with saturation, namely, the H-K effect, was observed for all uniform patch stimuli, a disk, rectangle, hand-shaped patch, and rectangular patch with two eyes and a mouth in the same manner as for facial images with a nonskin tone hue. However, brightness of all face stimuli in skin tone decreased with saturation, reflecting an inverse H-K effect even in scrambled images for *h*_ab_ = 60. As the stimulus size and shape of the uniform color patches were different from the face stimuli in Experiment 2, we queried brightness of Stimulus 3, which had the same size and shape as the face stimuli in Experiment 3. However, the results showed an increase in brightness with increasing saturation for Stimulus 3, as for Stimulus 1, which was the same stimulus used in Experiment 2. Therefore, both stimulus size and shape were irrelevant to the effect of saturation.

We could not confirm that face color hue was the factor that accounts for the uniqueness of face color perception, which could be sometimes considered the elemental purpose of human color perception. Our results showed similar effects of human skin tone hue saturation compared to any other hues in the uniform color patches. The commonality of the results in all hue conditions of the uniform stimuli suggested that the results might be explained sufficiently by specific bottom-up color perception mechanisms, such as cone sensitivity, the opponent color system, or specific color channels, such as the unique hues.

Furthermore, to investigate the effect of face recognition we also measured the effect of the saturation of the facial image in which only the hue changed. Brightness of the facial images for all hues except skin tone showed the same saturation effect as the uniform color patches, namely, brightness increased with saturation for all scrambled face images. Therefore, the inverse H-K effect was not observed by recognition of facial images with nonskin tones.

Although results from the debriefing interview after the experiment made clear that all the participants recognized Stimulus 2 (hand) as a hand and Stimulus 4 (rectangular patch with two eyes and a mouth) as a face, the results showed an H-K-like effect, as for Stimulus 1 (disk) and Stimulus 3 (rectangle), and recognition of a face or human skin was irrelevant to the effect of saturation. In contrast, brightness showed monotonic decreasing with saturation; that is, there was an inverted H-K effect for the face and even any spatial filtering of the scrambled face. Although half of the participants recognized Stimulus 6, “scrambled face (30 px × 30 px)” as patches of skin, the results of the debriefing interview showed that no participant recognized the image as a face. However, contrary to our expectation, the results of all the subjects were similar to those for the face image. Thus, the scrambled face image might have been perceived differently from a uniform color patch. Therefore, the recognition of a human face could not be the cause of the inverse saturation effect on brightness.

An alternative possible explanation of these results considers the uniformity of the images. High uniformity made the effect of saturation similar to the H-K effect, and low uniformity, such as in the face images, caused the inverse, namely, luminance changes affected brightness. In contrast to the uniform color patches, the dark elements representing the eyes, mouth, nose, and so on might lead to contrast, which could make brightness appear to increase. However, this hypothesis was not confirmed by comparing Stimulus 3 (rectangular patch) with Stimulus 4 (rectangular patch with eyes and mouth), or comparing Stimulus 5 (scrambled face without dark parts, 30 px × 30 px) with Stimulus 6 (scrambled face, 30 px × 30 px). In addition, brightness of the uniform color patches, such as Stimulus 3 or Stimulus 4, showed the same increment with saturation regardless of the presence of the dark parts, and brightness of the face stimuli, such as Stimulus 5 or Stimulus 6, decreased with increasing saturation regardless of the luminance variation.

In contrast, whiteness increased as saturation decreased, regardless of any shape or division of the stimulus image, because the color became closer to white as the saturation was lowered, as shown in Experiment 2. This implies that the monotonic decreasing of whiteness with saturation existed without image recognition. Therefore, the mechanism underlying whiteness perception could be independent of the mechanisms by which image types are perceived, and we could not observe any interaction between these two mechanisms. It should be noted that recognition of a face could not affect perception of whiteness. Perception of whiteness showed the different effect from brightness perceived not only by retinal information but also top-down cognitive information (Economou, Zdravkovic, & Gilchrist, 2015). This suggests that whiteness information is mainly obtained through a lower level neural mechanism, and little through any top-down information.

We also examined the sensitivity of perceptual judgments of brightness and whiteness of the uniform color patches and face stimuli by comparing the slopes of the functions. The slopes of the regression lines of brightness in face images were significantly steeper than those in the uniform color patches, as in Experiment 2, and steeper for the low-resolution faces (Stimulus 8, Stimulus 9, and Stimulus 10) than the high-resolution scrambled face images (Stimulus 5, Stimulus 6, and Stimulus 7). This suggests that the inverse H-K effect became salient in images of low uniformity, which rendered the image recognizable to the observer (*F*(1, 59) = 19.48, *p* < .0001). Brightness and whiteness in the face images could be assessed easier than in the uniform color patch as in Stimulus 3. Furthermore, the steeper slope of perceived whiteness than brightness showed that whiteness was judged easier than brightness in any image. Both of these results demonstrated reliability of the results from Experiment 2.

These results showed that perception of the scrambled face stimuli might have changed gradually in the experiment, although the inverse saturation effect on brightness, that is, the inverse H-K effect, was observed continuously. This gradual inverse change of the effect, depending on the special filter, was not observed for the other hues; that is, the shift occurred only in hues corresponding to skin tone. This suggests that the phenomenon of an inverse H-K effect in face brightness perception could be caused only by skin tones in a recognizably human face.

Adaptation or familiarity with skin tone is one of the most likely explanations for the difference in the effect of saturation on brightness between the uniform color patch and the face image. The participants in this study were Japanese females, and their facial skin tone has been reported to show relatively higher luminance (L*) and lower saturation (*Cc*), and vice versa. That is, whiter skin is also brighter skin in the faces they usually observe (Yoshikawa et al., 2012). These habitual experiences in daily observations might influence brightness perception of skin. Therefore, brightness of a face might correlate with its whiteness, namely, low saturation in the face image.

Primate color vision is characterized by trichromacy in which the wavelength sensitivity of the L and M cones is biased toward the long-wavelength range. This has been reported to be suitable for the detection of face color changes, unlike other types of chromatic perception, such as evenly spaced trichromacy (evenly distributed wavelength sensitivity of the three types of cones; Hiramatsu, Melin, Allen, Dubuc, & Higham, 2017). However, Chauhan, Xiao, and Wuerger (2019) recently found the negative results to this evolutionarily relevant hypothesis. This discussion suggests that the origin of our trichromacy is still controversial.

As noted earlier, approximately half of our participants noticed the images that displayed human skin in the experiments, even if they were unable to perceive the image as a face. It is possible that our neural mechanisms sample key features to create a prototype for perception under various illumination conditions. The inverted H-K effect was observed in only the stimulus images generated from face image regardless of observer’s awareness. Our visual system may be able to draw some important information from a partial, incomplete image without one’s awareness.

## Conclusion

We examined the effects of luminance on brightness and whiteness perception of faces and confirmed that both could be judged precisely following luminance variation in the first experiment. However, in Experiment 2, we found that perception of brightness of a face image was notably increased by decreasing saturation. That is, there was an inverse H-K effect at constant luminance, although brightness of the uniform color patch showed the H-K effect. This strongly suggested that the H-K effect is influenced not only by retinal bottom-up information but also by top-down information derived from recognition processes. Whiteness increased with decreasing saturation for both types of stimuli, indicating that whiteness is perceptually independent from brightness in face perception.

To investigate the reason for the unique nature of brightness perception for face images, we also explored effects of size, shape, specificity of skin hue, recognition of human skin, recognition of a human face, recognition of components of a human face image, and the luminance distribution. Moreover, as the effect showed the same slope direction as the participants’ face color distribution in the color space, we inferred that brightness perception of a face is dominated by the daily experience of the color range of human faces. This suggests the plasticity of human perception, in which brightness perception is stipulated by not only luminance but also other factors such as saturation. This affects the way in which decisions depend upon the perceived object and has developed during the process of human evolution. The results from our experiments might be limited only to images of faces because perception of real faces could be affected by additional factors, such as preference, expression, and familiarity. However, this study could serve as a basis for further research into the uniqueness of our perception as social beings, and as an example of plasticity in human perceptual processes.

An important implication of these findings is the flexibility and plasticity of our vision and the dynamic tuning of our visual system to the world. This study provides not only insight into color vision mechanisms but also useful information that may be applied in various industries.

## Authors’ Note

This research does not reflect the opinion that a darker skin tone is not beautiful. Certain Asian consumers have been encouraged to think that lightening their facial skin tone may make them more desirable. Market trends and past research have also reflected this. This study only seeks to examine what type of lighter skin tone is more desirable for that market: skin with light tonal pigmentation or white tonal pigmentation. This seeks to serve the market niche for Asian females looking to achieve their desired skin tone.
